# Co-production in local government: process, codification and capacity building of new knowledge in collective reflection spaces. Workshops findings from a UK mixed methods study

**DOI:** 10.1186/s12961-021-00677-2

**Published:** 2021-01-29

**Authors:** Peter van der Graaf, Mandy Cheetham, Sam Redgate, Clare Humble, Ashley Adamson

**Affiliations:** 1 Department of Applied Health Professionals, School of Health and Life Sciences, Teesside University, Middlesbrough, UK; 2 Department of Nursing, Midwifery and Health, Faculty of Health and Life Sciences, Northumbria University, Newcastle upon Tyne, UK; 3 Faculty of Medical Sciences, Population Health Sciences Institute, Newcastle University, Newcastle upon Tyne, UK; 4 Newcastle City Council, Newcastle upon Tyne, UK

**Keywords:** Co-production, Knowledge brokering, Translational research, Public health, Embedded research

## Abstract

**Background:**

Co-production of research evidence is valued by local government to improve effective decision-making about public services in times of austerity. However, underlying structural issues of power (so-called ‘dark shadows of co-production’) challenge this ambition with limited evidence on how to embed research use sustainably. In this paper we reflect on mechanisms for increasing co-production in local government.

**Methods:**

This paper presents findings from a Health Foundation funded research project that explored how a culture of evidence use to improve population health could be embedded in UK local government. Five linked work packages were undertaken using mixed methods. In this paper, we report the views of UK local authority staff who participated in four workshops (*n* = 54), informed by a rapid literature review and an online scoping survey.

**Results:**

We identified five themes that facilitate public health evidence use in local government: (1) new governance arrangements to integrate national and local policies, (2) codifying research evidence through local system-wide approaches and (3) ongoing evaluation of programmes, and (4) overcoming political and cultural barriers by increasing absorptive capacity of Local Authorities to embed co-produced knowledge in their cognitive structures. This requires adaptive governance through relationship building between academic researchers and Local Authority staff and shared understanding of fragmented local policy making, which are supported by (5) collective spaces for reflection within local government.

**Conclusions:**

Creating collective spaces for reflection in between government departments allows for iterative, interactive processes of co-production with external partners that support emergence of new governance structures to socially action the co-produced knowledge in context and build capacity for sustained evidence use.

## Background

### Co-production of research in local government

Local government in the UK is ideally placed to draw on and develop evidence to influence the upstream determinants of health and reduce inequalities. The National Health System in England is divided between commissioners (clinical commissioning groups) and providers (foundation trusts, community organisations and private companies) of health and social care services, with local authorities having a particular responsibility for public health. In their role as commissioners, local authorities are keen to co-produce services with service users and providers informed by the best available evidence, including academic research of (cost-) effectiveness.

Previous studies in the UK and internationally have highlighted various barriers for evidence use in policy making: research findings are often inaccessible to policy makers, they may value different types of evidence; and research timescales often do not align with policy processes [1–3]. Moreover, decision making is influenced by personal, social and political processes [4], in which tacit knowledge and other forms of evidence, such as local monitoring data, often trumps research evidence [5]. To overcome these barriers, several authors recommend closer interaction between public health practitioners and academic researchers [6]; however, progress remains slow [7].

At a time when austerity and public sector funding cuts have been factors limiting investment in research and evaluation in the UK, new models are needed to generate evidence through collaborative approaches, such as co-production of research between academics and local government staff. Co-producing research can be defined as “an approach in which researchers, practitioners and/or the public work together, sharing power and responsibility from the start to the end of the project, including the generation of knowledge” [8].

An example of such an approach are embedded research positions, which have shown promise in NHS settings [9], 10] and integrated care organisations [11] but are less well known in LG, with a few notable examples [12], 13]. These new models recognise the value of co-production of research evidence between academic researchers and professionals. However, these novel positions have also created tensions for the postholders, with lack of sustainable funding and short-term projects, making meaningful working together and power sharing challenging [10, 14].

### The challenges and politics of co-production

There is a rich and growing literature on co-production, with insight from the social sciences and humanities [15], political science [16], public management [17] and academic entrepreneurship [18] literature. The different frameworks and models discussed in this wide ranging literature all emphasise that the societal impact of research does not occur in a social or cultural vacuum, and is not simply transferred to society. Impact is realised in a network of interacting actors, interests and institutions, which takes time and effort to organise [15]. However, empirical studies on co-production are less frequent [19].

The focus of this paper is on the co-production of public (health) services, which aims to collaboratively produce knowledge involving academic researchers as well as LG partners to inform service development, with the active inclusion of all partners in the research design and process [20]. This approach is indebted to the work of Elinor Ostrom [21], who used the term co-production to describe a process through which ‘inputs from individuals who are not “in” the same organisation are transformed into goods and services’.

However, working in collaboration and co-production is not without its challenges. Recent studies reflecting on the value of co-production in research have highlighted the potential risks and costs, practical and professional, for both researchers and public health practitioners (PHP) who engage in this type of work. This so-called dark side of co-production [22] draws attention to the considerable time and resources needed to work in co-production, which can distract from other work (e.g. writing papers, funding application and teaching) and can create unproductive tensions due to power imbalances between researchers and PHPs, reducing trust and leading to poor quality research [22].

Critiquing the dark side-metaphor, Williams et al. [23] point out that co-production in itself is not inherently bad and that the shadow cast on co-production is caused by underlying structural issues of power (particularly in academic institutions). That is to say, the problem is not co-production itself but existing hierarchies and power relationships in a given context that stop co-production from working. The authors go on to argue that, if there is a dark side, it is the misuse of the concept of co-production. Therefore, we should not dismiss too quickly the feasibility and value of co-production of research evidence between researchers and PHPs.

Williams et al.’s critique echoes other studies in environmental science [24], which have shown that co-production can become the scapegoat for cost-cutting exercises and thorny political issues, “helping governments to abdicate their public responsibilities, while creating the appearance of consensus and shared responsibility between different social actors, and providing a convenient excuse for offering technical solutions to political problems”. Steen et al. (cited in [24] provocatively named these the seven evils of co-production: the deliberate rejection of responsibility, failing accountability, rising transaction costs, loss of democracy, reinforced inequalities, implicit demands and co-destruction.

### Context and process of co-production

To address these seven evils and making coproduction work, Williams et al. [23] argue that context is key: “the context in which co-production occurs largely determines the nature of the process and outcomes”. The authors claim that research provides a context for which co-production is often ill-fitted due to the power hierarchies and incentive structures in academic institutions.

Other authors have urged researchers and practitioners to be flexible and adaptive. For example, Pedersen, Grønvad, Hvidtfeldt [15] argue in their analysis of co-production theory and practice that researchers and societal actors can play many different roles in the co-production process at different times [15, 25]. At each stage of this process, different rationalities apply for what is considered best evidence, which modes of knowledge are preferred and how to assess its quality [26]. In other words, knowledge in co-production processes is constantly codified and re-codified in networks of interacting actors, interests and institutions. Knowledge is organised differently depending on the rationale for its use at different stages of the co-production process and the role that evidence users, producers and brokers play at each stage. Therefore, co-produced research is always an inherently political process involving negotiations between members of different “tribes”. The politics of co-produced research involves balancing very diverse interpretations as to what constitutes good research and impact [27].

### Governance in co-production

This points to another essential component of co-production: not only is it context-dependent, it is also realised in interaction, which requires *governance*. To this end Miller and Wyborn [28] introduced the concept of adaptive governance which they define as “iterative, interactive processes of knowledge production and sharing, planning, and action between different actors, networks and institutions”. Moreover, they contend that in these interactive processes, new patterns of governance and redistributions of power are produced that alter the balance between actors and introduce new roles and phases in the co-production process [29]. This makes knowledge co-production an inherently political process: “politics play an essential role in the remaking of knowledge and the use of knowledge is able to remake politics” [28]. To put it another way, co-production entails processes of reconfiguring science and its social authority.

This reconfiguring of authority is for Miller and Wyborn the real purpose of co-production: the goal is not to increase the use of research evidence in political decision-making or to blend different types of knowledge more effectively, but to create new forms of governance that produce the required knowledge and at the same time the social dynamics to act on it [28]. Contextually relevant knowledge and interaction processes, which define co-production, are contingent on the creation of new governance structures, which are themselves the result of these processes. This points to co-production as a complex system, which has also been highlighted by other authors [30, 31] who perceive the tensions and misalignments between research and practice as inherent complex system features that can never be eradicated.

The outcome of this complex process is sustained use of the co-produced knowledge and implementation of the altered governance arrangements within their local context. A structural factor influencing this outcome is the ability of knowledge users to embed the new knowledge in their organisations, practice and work cultures, which is summarised in the concept of absorptive capacity [32, 33]. The underlying idea is that organisations need the right (distributed) cognitive structures and learning capabilities in order to make full use of existing knowledge: organisations often lack expertise and a knowledge infrastructure for absorbing outputs of different knowledge transfer activities across different units both within and outside their organisation. We will come back to this point in our discussion and show an example of absorptive capacity in the form of collective spaces for learning between LG departments.

### A new definition of co-production in local government?

In short, co-production of knowledge can be simultaneously envisioned as a process, codification, and capacity: a contextual process of shifting roles and power balances that codify different types of knowledge at various stages in iterative and highly interactive structures governed by various actors, networks and institutions to sustainably embed the co-produced knowledge in their organisations and cultures by creating a shared language and capacity to absorb this knowledge. These three characteristics of co-production echo the building blocks identified by Denis and Lehoux [34] relating to the organisational use of knowledge, which they describe as knowledge as codification (focusing on the synthesis of knowledge in the form of clinical practice guidelines or quality indicators, which is one way of organising knowledge), knowledge as capabilities (in the form of organizational structures and processes to enable knowledge transfer) and knowledge as process (mechanisms to build relationships, create a greater sense of coherence and enhance problem-solving).

**Fig. 1:**
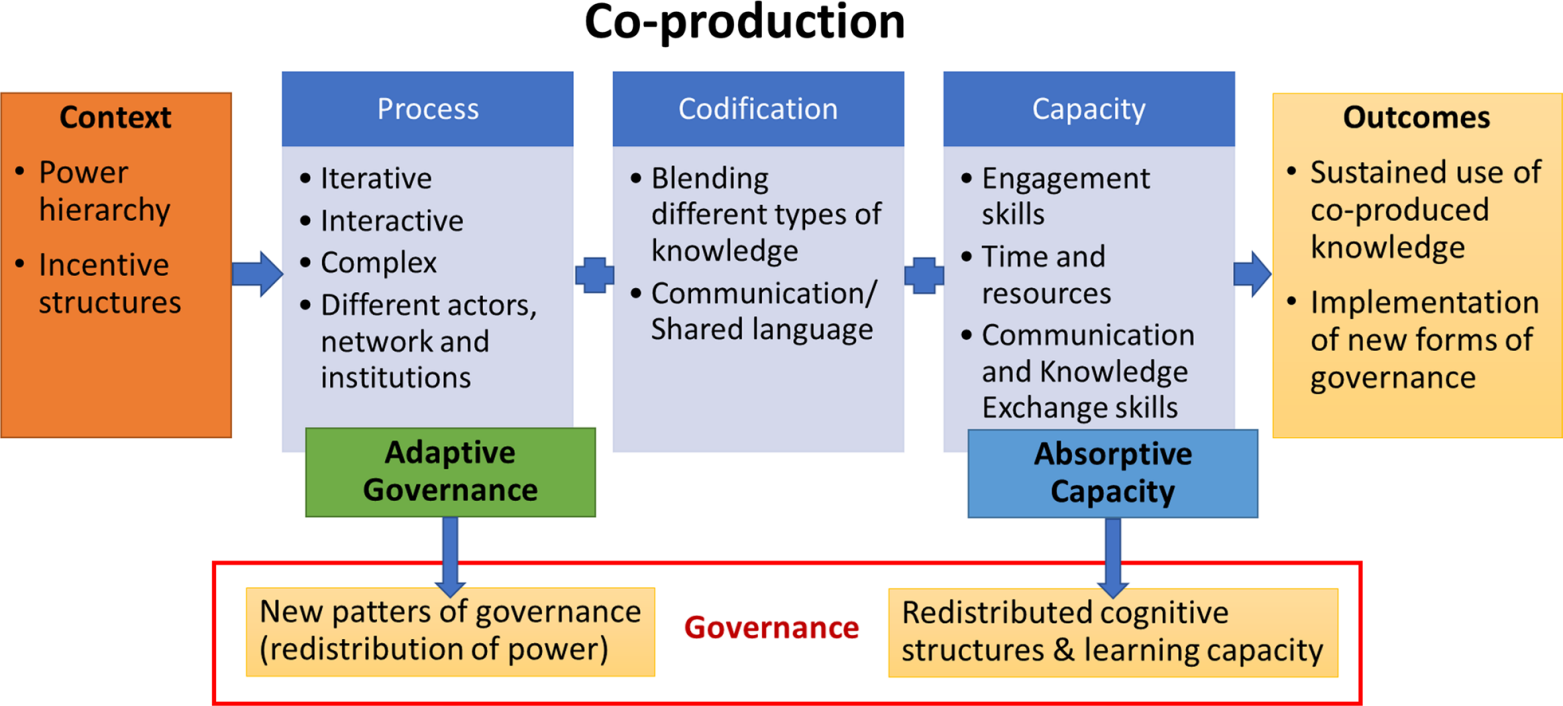
Model of co-production in local government

To these three building blocks of co-production we add the importance of context and the need for new governance structures to sustain knowledge use in LG, which are linked to the concepts of adaptive governance and absorptive capacity. This is summarised in our model below (Fig. 1).

We will use this model in our paper, to illustrate how collective learning spaces within LG can facilitate all three building blocks and support new governance arrangements within LAs and, therefore, provide a nurturing ground for co-production.

## Aims

This paper aims to define co-production of research evidence from a LG perspective by exploring the experiences of LG staff in three local authorities across the UK in co-developing, using and applying evidence in their commissioning of public health services. The perspectives are drawn from a Health Foundation funded study: Local Authority Champions of Research (LACoR) [35]. The study developed a proof of concept for embedding a culture of research and evidence use in LG focused on improving population health. By reflecting on their experiences we will identity underlying components of co-production, summarised in an emerging theory of co-production in LG, illustrated by finding from the LACoR study.

## Methods

The LACoR study consisted of five linked work packages using mixed methods to elicit views on evidence use and co-production from a range of stakeholders in LG. The study was conducted over a nine-month period between January and September 2019 with the findings from each work package informing the design of the data collection tools in the next work package to maximise data integration and facilitate an iterative research design.

We started by undertaking a rapid literature review on evidence use in LG, to identify common ‘headlines’ that informed the design of an online scoping survey. The survey was distributed to Directors of Public Health across England to identify different approaches to evidence-informed practice across LG. Based on the findings from the literature review and scoping survey, we facilitated workshops in three UK sites involving 54 participants to explore how evidence is currently used in LG, alongside the opportunities and challenges of using evidence in these three contexts.

The three LAs were selected from existing research partnerships with members of the research team to represent different LG structures for delivery of public health, representing a metropolitan council, unitary authority and a county council). The existing partnerships enable access to a range of participants in each workshop. All co-authors participated as facilitators in the workshops. In addition, a purposive sample of LA staff and stakeholders from one Local Authority (LA) were interviewed 1:1 (*n* = 14) by one member of the research team who worked as an embedded researcher in the LA to explore perceptions of what, why and how evidence is currently used in LG in more detail.

The combined data informed the development of a logic model (see Additional file 1) which was tested and refined in two further workshops with stakeholders in Rivertown (*n* = 13) and nationally (*n* = 27). Ethical approval for this study was obtained from Newcastle University (ref no. 48424) and written informed consent to participate was obtained from all study participants. The names of the study sites have been changed in this paper to ensure anonymity of reporting.

In this paper we report on the findings from the workshops, as these sought to identify mechanisms for improving evidence use and, in doing so, provided reflections on co-production practices in LG. Written notes by table facilitators and flipcharts from each of the table discussions at each workshop were typed up and analysed thematically by the research team using a using a coding framework informed by the topic guide for the workshops (see Additional file 2). Themes were coded inductively initially and then mapped onto the six components of our model for co-production in local government (Fig. 1) to assess fit and inform interpretation of the identified themes, on which we reflect in our discussion section, and to aid the development of recommendations for improving co-production of research evidence between academics and LG staff, which is summarised in our conclusion the end of this paper. As the workshops were not recorded, very limited direct quotes were available for analysis, although we have included one quote of a workshop participant at the end of this paper.

Findings of other work packages, including the scoping review, the interviews and the logic model are currently being written up for reporting elsewhere. More details on each work package and their findings are also available from the Health Foundation report [35]. Below, we provide more detail on the structure and participants in the workshops before we present the findings of the workshop discussions.

### Facilitated workshops

Following an initial workshop in Rivertown, subsequent workshops were held in Belltown and Castletown attended by 54 participants in total (more details on participants in each workshop can be found in Table 1). Topic guides were used to focus discussions on different topics identified by PHPs in each workshop: ‘school readiness’ in Rivertown, ‘health inequalities’ in Belltown, and ‘health in all policies’ in Castletown. Facilitated workshop discussions provided an opportunity to scope local needs in relation to the chosen topic and examine the existing networks that drive decision making and use of evidence. In each workshop, we explored beliefs about the value of research evidence, the value of different models for evidence-informed decision making, the routine application of existing evidence and participants’ views on the potential for co-creation of new evidence that would address their challenges and priorities.

**Table 1:** Description of participants in each workshop (*n* = 54)

Workshops	Participants roles	Total
Rivertown	Senior Specialist—Public Health (Children and Young People), Public Health Intelligence Specialist, and Service Manager—Early Help and Family Support, Performance Analysts, Information Managers	14
Belltown	Representatives from Belltown City Council, Belltown HSC Trust, Health and Social Care Board, South EHSC Trust, Libraries NI, Belltown Health Development Unit, West Belltown Partnership Board, Northern Ireland Housing Executive	22
Castletown	Representatives from Hampshire County Council and Castletown City Council, including Assistant Director, Internal Provision and Front Door, Head of Insight and Engagement, Head of Research and Intelligence, Head of Corporate Customer Service and a newly appointed embedded researcher. Academics in Public Health and Medicine from Castletown University	18
Rivertown (2^nd^/ follow-up workshop)	Participants included: Senior Public Health Specialists, Insights Manager, Performance Analysts, Service Improvement Leads, Community Safety Specialist, Communities Officer, Policy and Communications lead, Public Health Intelligence Lead, two Directors and a voluntary organisation chief executive	

Although each authority chose different public health topics to frame their discussions, workshops were facilitated using similar prompt questions. The findings were then presented at a fourth workshop by means of a ‘sense check’, generating group discussion among LA participants (*n* = 13) regarding the appropriateness of the logic model along with practical considerations of its application.

### Workshop structure

Each workshop started by exploring the chosen topic by LA stakeholders in more details, to get participants thinking about the topic area and to clarify terms, for example, in Rivertown the initial discussions focused on how participants defined school readiness. This discussion was followed by a future visioning exercise, asking participants to imagine that it’s 10 years later (2029), with their chosen subject area completely embedded in LG and achieved in their local area (e.g. and all children in Rivertown are ready for school) and asked to note down on post-it notes what three things pleased them most and what had happened to ensure this. This exercise was designed to help participants define outcomes in relation to their topic area. Participants were then asked to collectively cluster individual notes in groups on a flip chart. In this paper, the results from this exercise will be compared across workshops to illustrate how LA staff would like to use and co-produce research in LG.

The future visioning exercise was followed by table discussion on current practices for using evidence (e.g. statistics, performance measures, research, local intelligence) in their chosen subject areas (e.g. how do you know that a child is ready for school?) and to inventory where this evidence was coming from; e.g. what information sources were used to evidence their selected outcomes? This discussion also aimed to get an understanding of who had access to this evidence and who held and used what data.

In the follow-up table discussions, participants looked at what helps and hinders evidence use in decision making and how evidence use could be improved. Finally, in the last set of table discussions, participants were asked about the potential role of research in the LA, particularly who and what was currently missing to achieve this. This session aimed to get a better understanding of what types of evidence may be missing and what the strengths were of inter and intra-organisational relationships to support evidence use.

## Results

Although each workshop focused on a different topic chosen by participants in advance, there were similarities in the themes emerging from each workshop, which point to significant potential triggers for evidence use in LG. The five overarching themes were:

- Aligning national and local policies
- Local system-wide approaches
- Evaluation of local programmes
- Political and cultural barriers
- Collective spaces for reflection

We will discuss each theme separately below with illustrations from the four workshops and link each theme to one of the six building blocks in our model of co-production in local government (Fig. 1), which envisions this concept simultaneously as a (1) process, (2) codification, and (3) capacity: an iterative and highly interactive process of shifting roles and power balances between LG staff and academic researchers that codifies different types of knowledge at various stages in this process, which requires capacity within LG to absorb the co-produced knowledge and embed it in their cultures by creating a shared language. In addition, our model highlights the importance of (4) context and the need for new governance structures to sustain knowledge use in LG, which are linked to the concepts of (5) adaptive governance and (6) absorptive capacity.

### Aligning national and local policies

In all three workshops, participants differentiated evidence at different spatial levels; often starting with national government and the implications of policy development at this level for statutory data requirements at the local level. In each workshop, participants emphasised the need for a change in policy focus and related outcomes, and more integration of national policies across different government departments, to be able to change their own focus in local outcomes and data needed to account for these outcomes.

For example, in the Rivertown workshop participants discussed a definition of school readiness that incorporated two broad categories: academic skills and social skills of children at age 5. In the table discussions, acquisition of social skills (and a broader definition of skills for life) emerged as the more dominant category and preferred way to define school readiness. Participants concluded that social and soft skills were most important in order to create an individual environment in which the young person could thrive. However, when reviewing the current evidence that was being collected within the LA on school readiness, indicators for academic skills were much more prevalent. For instance, Early Years Foundation Stage Profiles at end of reception, the Ages and Stages Questionnaire, pre-school assessments all tended to focus on cognitive and language skills.

Moreover, these data were collected by different organisations, such as schools, health visitors, nurseries and including private providers, which made it difficult to compare and data was often not shared between organisations or only available at population level. Many of these data collection processes were dictated by national government as part of the statutory delivery and monitoring of services and, therefore, LAs reported minimum wriggle room in deciding what data to collect because of these national mandated targets and practical challenges surrounding data sharing. In sum, LAs were collecting data which did not match their preferred definition of school readiness to inform local policies, and were not able change this.

### Local system-wide approaches

The need for more integration of policy and data collection was repeated at LG level with participants advocating for closer collaboration between LG departments on data sharing, pooled budgets and integrated services. Adopting a system wide approach to improving health that included other LG departments and facilities, such as transport, sports and leisure, was seen by participants as more effective in achieving outcomes. Getting the right people around the table from different local departments and aligning research to political language, was deemed as essential. For example; the word ‘intervention’ means very different things to a researcher and a local councillor, with the latter often assuming a negative connotation, such as children being taken into the care of the LA.

The need for co-production as an integration process, which codifies knowledge for different audiences, was also extended to wider partners, including NHS and VCS organisations. Participants across all three workshops pointed out the need to work closely with external partners outside their LA, such as schools, parents and the wider public (process). Communication was seen as key for this and, therefore, evidence needed to reflect the objectives and needs of these partners by answering the question ‘what is in it for them’ (codification). Moreover, partners needed to be confident and knowledgeable to engage and therefore dialogue was considered important.

Codification in co-production was deemed particularly relevant at the community level, by giving local communities a greater voice in decision making and resource allocation. More community ownership of services was seen as better way of targeting those that need the services the most by enabling communities to design where and how services would be delivered. Research data should support and reflect this by highlighting areas with the greatest inequalities and service needs. Not surprisingly, health and social inequalities were seen as key focus for local and national policies.

In the Belltown workshop, participants mentioned an ongoing struggle to incorporate community data (described as soft intelligence from these communities) in their decision making, which they were keen to do. They recognised the importance of a community voice in their decision making in order to target areas of highest need. However, the LA culture of evidence in Belltown prioritised quantitative survey data (e.g. from the national Statistics and Research Agency), which they were trained to use, over qualitive data from local communities, which participants did not know how to access or use effectively. In other words, the LA lacked the absorptive capacity (see Fig. 1) [32, 33] to use the knowledge from communities as it was not codified in way that the LA could embed in their cognitive structures. These cognitive structures depend on effective communication and a shared language between the different co-production partners; in this case, Belltown LG staff and representatives from local communities.

To create this shared language, participants in the Belltown workshop suggested a codification based on the concept of social and economic inequalities in all policies. This would facilitate a move away from policies for the ‘average person’ (which is the focus of quantitative survey data) and instead advocated targeted policies for more deprived areas for which qualitative data is more appropriate, as it provides richer insights in specific cases.

### Evaluation of local programmes

Participants agreed that a different codification of knowledge required a different way of collecting, collating and using evidence within LG. All three workshop participants expressed a need for ongoing assessment and evaluation of programmes within LG, which they felt was currently lacking. These assessments and evaluations were essential to answer different research questions: What worked well for whom and where? How can we make better use of existing data within LAs, including survey data and population statistics, combined with qualitative data? To support effective and relevant evaluation, participants suggested the need to build in reflection and evaluation from the start of new programmes: thinking through how a programme might work and understanding key outcomes and, therefore, what evidence is needed to assess these outcomes to demonstrate a change in health outcomes and the effect of a programme on health inequalities.

Currently, participants complained that fragmentation in LG and silo-thinking between departments hindered data sharing and joint learning: different professional cultures within LG and, in some cases, a lack of an organisational culture that supports research use was not conducive for collecting, collating and using evidence within LG to facilitate more programme evaluations and assessments.

For example, in the Castletown workshop participants reported a lack of incentives within their LA to evaluate local programmes and services. What was valued by programme leaders and commissioners was the delivery of projects and not their evaluation. Incentives and performance targets for projects were based on delivery of the service (outputs and numbers) and not what difference the service made to the health and wellbeing of service users. Evaluations could even be counterproductive: some Council staff admitted that they did not value evaluations, as they did not want to hear what did not work in their project, which could endanger future commissioning and funding of the service.

Similar to the participants in the Belltown workshop, participants in Castletown identified that their LA lacked the absorptive capacity (see Fig. 1) to use research evidence. In this case not because the evidence was not codified in a fitting way with LG decision making processes but because the cognitive structures were not able to absorb any research evidence. Perverse incentives caused a culture of anti-evaluation with a complete lack of willingness to undertake co-produced research.

### Political and cultural barriers

What emerged from the three workshop discussions was a deeply politicised and fragmented system in LG that put different demands and constraints on evidence use, depending on the context and decision making process in which evidence was used. The political system was described as disorderly by participants, but also as opportunistic in nature. Participants highlighted that there were opportunities in this disorderly system to insert research evidence into decision making processes at the right time.

To maximise these opportunities, researchers need to understand the wider social and political systems in which these processes operate and utilise contextually specific knowledge to identify levers of influence. This includes acknowledging that there are multiple sub-cultures of evidence use within the council; each with their own policies and legislation. Understanding the timings of the political process (e.g. four yearly election cycles with peaks and troughs and different windows of policy making) and identifying trusted contacts in LG that could act as the ‘go-to-person’ for different cultures, were seen as essential pre-requisites for researchers.

What emerged from the workshops were very different cultures of evidence use in academia and in different departments in LG. Participants thought that academic research was geared towards academic outputs: with researchers prioritising papers in peer reviewed journals and large funding applications to prestigious research programmes, for which local evaluations were often not a good fit. On the other side, LG professed to a culture of risk aversion not conducive to academic research, as negative evaluation findings could endanger future commissioning and funding of a service, and a prioritisation of front line delivery where time is of the essence and academic research often takes too long.

### Collaborative spaces for reflection

Workshop participants kept turning back in the table discussions to a lack of collective shared spaces for reflection. In the daily rush to support frontline delivery of services with a lack of resources, participants complained that no time and space was available to look at their commissioning plans and reflect on desired outcomes and how best to deliver them, informed by research evidence.

According to participants, this problem was exacerbated by silo working within different government departments, each with their own policies and culture. This fragmentation is perpetuated by national government mandates around statutory deliverables and data collection, which structures different outcomes in different silos of LG (that are not always shared across departments). These silos will need to be integrated first before a joint-up research culture can be embedded in LG. Some participants suggested temporary suspension of statutory legislation within LG to enable this; however, this is unlikely to be realistic option, at least in the short term.

Therefore, participants were keen to create spaces for reflection within LG, not just in one department, as this would potentially sustain the existing silos, but by creating spaces between departments for collective reflection. These spaces were envisioned as group of people from different departments coming together on a regular basis to share knowledge and training, to reflect on the evidence and knowledge that they use, and to make connections between people for mobilising knowledge across LG. These spaces could also include wider collaborations with external partners, such as the police to discuss what they do and what evidence they use. Examples of these spaces can already be seen in some local community safety partnerships but this approach could be adopted wider across the council in other topic areas.

## Discussion

Recent studies of organisation-wide improvement initiatives [35] have shown that the creation of a positive, collaborative and inclusive workplace culture, including a learning climate with time and space for reflective thinking, can be associated with more evidence use and improved patient outcomes [36], which in turn can improve staff morale, retention and quality of services. These initiatives recognise and draw together different forms of knowledge from multiple sources, including political actors, enabling collective wisdom and insights to be generated alongside an understanding of contextually relevant solutions.

However, little evidence is available on how to implement these practices into LG and how a research culture can be embedded across different LA that takes account of the ‘dark shadows of co-production’ [22], in particular underlying structures and power dynamics that hinder the use of research evidence in LG. Our paper contributes to the existing literature on co-production by highlighting from an empirical example what these new governance arrangements can look like. Much of the current literature is concerned with conceptual models and frameworks [15] with a lack of empirical studies that illustrate how to apply these models in practice [19]. Our model, based on recent discussions in this journal about the dark side and shadows of co-production [22, 23], identified six building blocks of co-production (process, codification, and capacity, context and adaptive governance/ absorptive capacity). We applied this model to our workshop data to illustrate how collective learning spaces within LG can facilitate the different building blocks and support new governance arrangements within LAs and, therefore, provide a nurturing ground for co-production.

### Summary of key findings

#### Co-production as a process: data integration between national and local policies

Firstly, participants highlighted the need for co-production as an integration process [28] (see Fig. 1) of national and local policies and associated data collection. National governance arrangements around statutory data requirements hindered focusing local policy efforts on identified priorities and the sharing, collating and use of relevant data on these priorities across different departments. Moreover, the wider system outlook for evidence use that emerged from the workshops is important to consider for researchers. Research evidence does not only need to be made fit for LG decision making but needs to fit in at different levels and for different organisations. This requires a degree of flexibility and adaptability of research findings that is hard to achieve by researchers alone. Co-production of research evidence is one way that participants have suggested for building in this adaptability of research.

#### Co-production as codification and capacity: learning the politics of local decision-making and including community voice

Adapting research evidence and aligning data between national and local policies points to the second building block of co-production in which research evidence is codified [15] (see Fig. 1) to make it fit with the political context and agenda in which it is to be used. Researchers need to understand how decision-making actually works and learn the politics of the local decision-making processes. Previous research [37] points to the limitations in readiness of researchers to work in the fast-paced policy and practice environments. Skills are required in political sensitivity, negotiating, influencing, persuasion, change management, problem solving, teamwork and leadership [38]. Therefore, developing capacity (see Fig. 1) and capability to engage through training, both for researchers to engage confidently with LG and for LG staff to understand their communities’ needs. Codification in co-production was deemed particularly relevant at the community level, by giving local communities a greater voice in their decision making and resource allocation.

However, LAs often lacked the absorptive capacity [32] (see Fig. 1) to use the knowledge from communities as it was not codified in a way that the LA could embed in their cognitive structures. These cognitive structures depend on effective communication and a shared language between the different co-production partners, in this case LG staff and communities. Codification of research evidence and community voice allow for flexibility and adaptability of the co-production process and enable researchers and LG staff to play different roles at different stages of this process ([15].

#### Co-production as capacity to absorb new knowledge through evaluation

Fourthly, in some LAs the issue was not a lack of codification but a lack of willingness to engage in co-produced research because their internal cognitive structures were not always able to absorb the co-produced evidence. Perverse incentives caused a culture of anti-evaluation with staff actively resisting evaluation of commissioned programmes and services. The context within LAs did not fit the co-production process, as problematised by Williams et al. [23]. These findings are similar to previous research [37] highlighting that senior health managers see research and evaluation as separate activities in their organisations that coordinated by different responsibility areas.

Therefore, most participants expressed a need for ongoing assessment and evaluation of programmes within LG to enable a different codification of knowledge by answering different research questions (what worked well for whom and where?), making better use of existing data and local intelligence within LG, combined with qualitative data, and building in reflection on key outcomes and evidence needs from the start of new programmes.

#### Co-production as adaptive governance: ongoing relationship building through collective reflection spaces

Fifthly and most importantly, participants emphasised the need for adaptive governance [28] (see Fig. 1) to overcome political and cultural barriers, with ongoing relationship building between academic researchers and LG staff based on a shared understanding of the deeply politicised and fragmented system of local policy making. While appearing chaotic, participants were adamant that this system presents opportunities to weave in accessible evidence locally, which requires contextually specific knowledge. It is vital for academic researchers to build their capacity to engage effectively in this process through communication skills, knowledge exchange expertise and understanding of LG systems.

Previous studies [39], 40] have highlighted the valuable role that boundary spanners and knowledge brokers can play in in translating research evidence by acting as ‘evidence champions’ [10] and ‘credible intermediaries’. In our study, we found that these roles already existed in various departments across LG; however, these roles and the people fulfilling them were underutilised and could be used more effectively in co-production evidence between academia and LG.

#### Oiling the co-production model: collective reflection spaces in LG

A key ingredient for adaptive government arrangements is the creation of collective reflection spaces in LG for iterative, interactive processes of knowledge production and sharing, planning, and action between different departments and multi-agency partners. These spaces allow for the emergence of a new governance structure, echoes the characteristics of adaptive governance set out by Miller and Wyborn [28] that can overcome co-production barriers, such as insufficient codification of research evidence and local knowledge and a lack of cognitive structures to embed the new co-produced knowledge. These spaces build on previous suggestions in the literature [41, 42] for embedded research—a collaborative, adaptive approach to improvement, which involves researchers and implementers working together in situ from the outset of, and throughout, a project.

What our research add to this literature is a need to embed this process within local government and between different departments to fit in with existing power hierarchies [23] but also to redistribute power through these spaces. Collective learning spaces alter the balance between researchers and LG staff, introducing new roles and phases in the co-production process [28]. These new forms of governance not only produce the required knowledge but at the same time also create the social dynamics to act on this knowledge.

### Strengths and weaknesses of this study

Our mixed methods approach, and the involvement of multiple stakeholders, including representatives from universities and LG across the UK add depth and understanding to the issues raised by LG staff and academic researchers. We recognise that, although geographically, culturally and politically distinct, the three participating LA may not be representative of all LA and were opportunistically selected from existing research partnerships within the research team. In the short timescale of this study, we were not able to capture the views of elected members, those outside LG, in funding bodies or among academics with experience of co-production in LG. We also did not include the views of service users or other members of the public, as our study focused on the perceptions of LG and their partners, which often did not include community voices as highlighted in our findings. Moreover, our collected data from the workshops lacked a richness in detail with the absence of quotes, as we did not record the workshop discussions and instead had to rely on written notes from facilitators and flipcharts. Despite these limitations, our analysis of the workshop data provided new insights and our findings are in line with previous studies, which suggests a degree of validity of the results.

All workshop facilitators were based in universities and this may have affected the responses of participants. It was partly in response to potential bias that we also held a follow-up workshop in Rivertown to sense check our findings with practitioners and a national workshop with participants from all three LA, which offered opportunities for in-depth discussion of the issues and allowed the emergence of consensus on these issues, as well as discussion that was solution-focussed to overcome dark shadows of co-production and embedded research in LG.

## Conclusion

In this paper, we sought to identify and elaborate on the underlying components for making co-production in LG work, based on the findings from a Health Foundation funded research project (LACoR) that explored how a culture of research and evidence use to improve population health could be embedded in LG.

In our study we found a clear appetite among LG staff to use research evidence in their decision making; however, in practice research evidence was not routinely used in their work. Research evidence was often not deemed relevant because it did not ask the right questions or fit with the context, timeframes and policy process in which it was needed. Co-production of relevant, timely and useful research evidence between academic researchers and LG staff was suggested as an important mechanism for facilitating more evidence use in LG decision making.

However, the politics of co-production are often ignored by academic researchers, resulting in counterproductive outcomes: creating unproductive tensions, reducing trust and leading to poor quality research. Instead, we have argued in this paper that successful co-production is context dependent and highly interactive, which requires governance and therefore makes co-production a political process. The workshop findings with LG staff (*n* = 54) in three localities across the UK on research use in decision making highlight that new governance arrangements are needed in the form of collective reflection spaces that facilitate co-production of new knowledge and its enactment in local organisations. One workshop participant framed these spaces as follows:

“Think of the research function as an essential element of a multidisciplinary team. The real transformational benefits always go both ways—it needs an ongoing commitment and understanding that in order to both benefit a pooling of resources is necessary (i.e. matched funding arrangement)”.

Workshop participants emphasised that collaborative research is a contextual process and therefore the governance arrangements need to reflect a range of local partners within LG (different departments) and outside (private service delivery, community). This highlights the need for complementary expertise in communication and knowledge brokering among academic researchers and LG staff but also for spaces for both to come together for reflection.

Participants accepted that creating collective spaces for reflection would have resource implications to allow staff time away from their desk to reflect with peers. Moreover, to make these spaces possible a less strict focus on service delivery is required. Participants argued for a change in the performance management structure within LG, away from national statutory outputs to locally produced targets that make sense from a community perspective (see Belltown case study).

In other words, to enable the collective space for reflection the cognitive structures within LG need to change first by increasing the absorptive capacity within the organisation (change in performance management structure) and by building capacity for engaging in research through an overarching corporate research policy. This structural change is only possible with senior level buy-in, which requires a codification of the knowledge reflected on in these spaces by political agenda setting.

Based on our participants’ suggestions for embedding collective reflection spaces in LG, we make the following recommendations to encourage the process, codification, and capacity of knowledge co-production, which is sensitive to political context and power hierarchies, and supports new governance structures to sustain knowledge use in LG, by creating a shared language and capacity to absorb this knowledge.

We hope future research will be able to look at the transferability of this model to different local authority contexts in the UK (e.g. county councils; district, borough or city councils; and one tier/unitary councils) by comparing the co-development, implementation and the research evidence mobilised in these spaces over time across different areas.

### Recommendations

Practical

- There is a need for complementary expertise in communication and knowledge brokering among academic researcher and LG staff but also for spaces for both to come together for reflection
- Future job descriptions for staff in LG could include a requirement to spend 20% of their time in a different directorates to foster collaboration and understanding across LG

Research

- Co-production facilitates adaptability of research: by working closely from the start in the research process with partners and evidence users ensures that findings are relevant, timely and tailored to the local context in which it is needed
- It is vital for academic researchers to build their capacity for engaging effectively in co-production processes with LG through training in communication skills, developing their knowledge exchange expertise and increasing their understanding of LG systems

Policy

- New governance arrangements for co-production need to reflect a range of local partners within LG (different departments) and outside (private service delivery, community)
- Reflective spaces for developing these arrangements require an overarching corporate research policy and LG-wide service to coordinate and encourage reflection and research between departments
- To ensure buy-in for these spaces, the agenda for the network should be set by the political leadership in LG (making it a political network) in order to find a balance between relevance and rigour. Therefore, engagement with elected members and chief officers is important to make the issue politically relevant

## Supplementary Information

**Additional file 1.** Logic model.

**Additional file 2.** Topic guide for workshops.

## Data Availability

The datasets used and/or analysed during the current study are available from the corresponding author on reasonable request.

## Abbreviations

LA: Local authorities
LG: Local government
PHP: Public health practitioners
