# Supplementary figures and images for: Co-production in local government: process, codification and capacity building of new knowledge in collective reflection spaces. Workshops findings from a UK mixed methods study
Source: Health Res Policy Syst. 2021 Jan 29;19:12. doi: 10.1186/s12961-021-00677-2 (PMC7844986; doi:10.1186/s12961-021-00677-2)

**Supplementary File 1. Logic model from the Local Authority Champions of Research (LACoR) study**


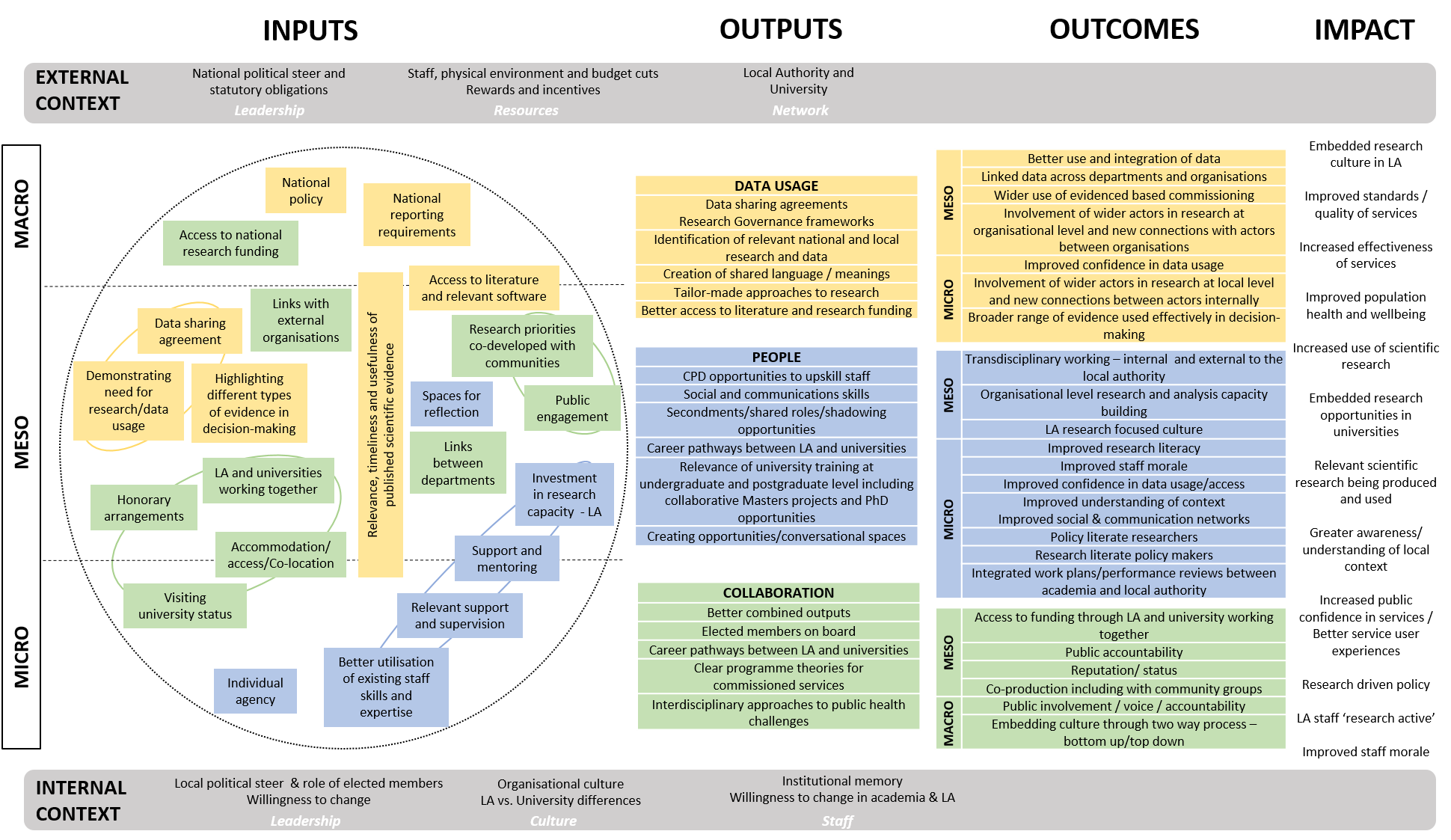

Supplement: Supplementary file 1 — Additional file 1. Logic model. [file 12961_2021_677_MOESM1_ESM.docx]
