# Supplementary material for: Co-production in local government: process, codification and capacity building of new knowledge in collective reflection spaces. Workshops findings from a UK mixed methods study
Source: Health Res Policy Syst. 2021 Jan 29;19:12. doi: 10.1186/s12961-021-00677-2 (PMC7844986; doi:10.1186/s12961-021-00677-2)
Supplement: Supplementary file 2 — Additional file 2. Topic guide for workshops. [file 12961_2021_677_MOESM2_ESM.docx]

## **Supplementary File 2.**

**Local Authority Champions of Research (LACoR) workshops programme and prompt sheet**

| **Time** | **Content** |
| --- | --- |
| 09:00 – 09:15 | **Arrivals**  Arrivals and refreshments  Registration  PIS and consent forms distributed  Set up presentations |
| 09:15 – 09:45 | **Welcome and introductions:**   - Welcome and introductions - Introduce research team and explain aims of workshop - Request consent forms - Background, rationale for choice of topic and future opportunities - Participants introduce themselves (name, role, length of service in council) - Working agreement |
| 09:45 - 10:05 | **Understanding school readiness**   - How would you define School readiness? What do we mean when we talk about school readiness?   - Write responses down on flip chart paper following discussions in pairs   - *AIM: get respondents thinking about the topic area and clarify terms* |
| 10:05 – 10:35  10:35 – 10:45 | **Future visioning**   - The year is 2029, and all children in Newcastle are ready for school. What three things have happened to ensure all children in Newcastle are ready for school? What if…?   - Write responses down on post it notes and stick on flip chart paper on the wall   - *AIM: to set scene, engage participants and define outcomes in relation to topic area*   Group feedback from each table (5 mins each) |
| 10:45 – 11:00 | ***Comfort break*** |
| 11:00 – 11:30  *10 mins per question* | **Understanding current practice**   - How do you know that a child is ready for school?   - Write responses down on flipchart paper – specific examples   - *AIM: Gauge participant’s understanding of school readiness and what influences it* - What evidence/statistics/performance measures/research are collected to show school readiness?   - Write each down on separate post-it note then group discussion   - *AIM: Get an understanding of what information sources are used to ascertain school readiness and by whom* - Where does the evidence/statistics/performance measures/research come from?   - Put the post-it notes into groupings (on flip chart paper) dependent on where the information comes from   - *AIM: Get an understanding of who has access to and who holds what data* |
| 11:30 – 12:00  *10 mins per question* | **Understanding current use of research**   - How is the evidence/statistics/performance measures/research used and by who (role not name)?   - Refer back to the post-it notes from Q3 and use separate flip-chart page to write down how each is used and by whom   - Prompt if required on; commissioning decisions, performance targets, performance measures   - *AIM: Understand how research is currently used* - What helps and hinders evidence/statistics/performance measures/research to be used in decision making?   - Write responses down on flip chart paper   - Prompt if required using responses from Q5   - *AIM: Understand current practices, perceived barriers and facilitators* - What could be improved in terms of the use of evidence/statistics/ performance measures/research?   - Write responses down on flip chart paper   - Prompt if required using responses from Q5 or on examples of other areas where it has worked well. Explore who would lead areas/actions suggested   - *AIM: Understand current practices requiring improvement* |
| 12:00 – 12:20 | **Understanding potential role of research**   - Who and what is missing?   - Prompt on:     - What evidence is missing? Are there any gaps in our knowledge / understanding?     - What else is needed?     - Who else needs to be involved in discussions / plans     - Any risks / unintended consequences of focusing on school readiness?     - What else might affect efforts to introduce change at the present time   - Write responses down on flip chart paper   - *AIM: Get an understanding of what types of evidence may be missing and strength of inter/intra-organisational relationships* |
| 12:20 – 12:30 | **Close and thanks**  Round up, next steps and final round of feedback – one thing from the afternoon that will stay with you or make you think differently. |
